# The mediating role of learning self-efficacy in the relationship between subjective well-being and academic performance in children

**DOI:** 10.3389/fpsyg.2025.1570068

**Published:** 2025-04-25

**Authors:** Tingyu Zhang, Xiang Chen

**Affiliations:** ^1^School of Humanities and School of Design, Jiangnan University, Wuxi, China; ^2^School of Design, Jiangnan University, Wuxi, China

**Keywords:** teenagers, subjective well-being, learning self-efficacy, mediating effect, academic performance

## Abstract

**Introduction:**

Academic pressure can significantly impact the mental health and overall well-being of children. This study investigates the relationship between subjective well-being and academic performance, with a focus on the mediating role of learning self-efficacy.

**Methods:**

Data were collected through questionnaire surveys administered to a sample of 1,022 children from seven schools in City A. Statistical analyses, including Pearson correlation and structural equation modeling using the Bootstrap method’s Model 4, were conducted to examine the direct and indirect effects of subjective well-being on academic performance, with learning self-efficacy as a mediating variable. The influence of demographic factors, such as family structure and upbringing, on subjective well-being and learning self-efficacy was also explored.

**Results:**

The findings demonstrate a significant positive relationship between subjective well-being and academic performance (*r* = 0.343–0.351, *p* < 0.01). Mediation analysis revealed that learning self-efficacy partially mediated this relationship, with direct effects of subjective well-being on academic performance (*B* = 0.24, *p* < 0.001) and indirect effects via learning self-efficacy (*B* = 0.46, *p* < 0.001; 95% CI [0.33, 0.48]). Additionally, demographic factors, such as being an only child (*t*(1020) = 2.69, *p* = 0.008), being raised by both parents (*t* = 2.79–3.56, *p* < 0.001), and urban/rural upbringing (*r* = 0.35, *p* < 0.01), were significantly associated with both subjective well-being and learning self-efficacy.

**Conclusion:**

This study underscores the dual pathways through which subjective well-being influences academic performance in children: directly and via learning self-efficacy. Practically, these findings advocate for targeted interventions to enhance children’s mental health and learning self-efficacy, such as integrating resilience-building modules into school curricula and training educators to recognize early signs of low well-being. Additionally, the findings highlight the importance of considering demographic factors in educational planning and policy-making to further support students’ academic success.

## 1 Introduction

In recent years, socio-cultural shifts—such as rising divorce rates, urban–rural educational disparities, and family dynamics—have profoundly influenced children’s learning environments and mental health (Ortúzar et al., 2021; Cattelino et al., 2021). These contextual factors underscore the need to investigate mechanisms linking psychological well-being to academic success. Empirical studies consistently demonstrate a positive association between subjective well-being and children’s academic performance (Liu et al., 2021; Iqbal, 2022). For instance, Li et al. (2023) found that higher life satisfaction predicted better grades among Chinese adolescents, while Koca et al. (2024) identified learning self-efficacy as a key mediator in this relationship. Furthermore, Diotaiuti et al. (2021) highlighted the mediating role of emotional balance and procrastination in academic performance, emphasizing that emotional well-being significantly influences students’ ability to manage academic tasks effectively.

**Figure 1:**
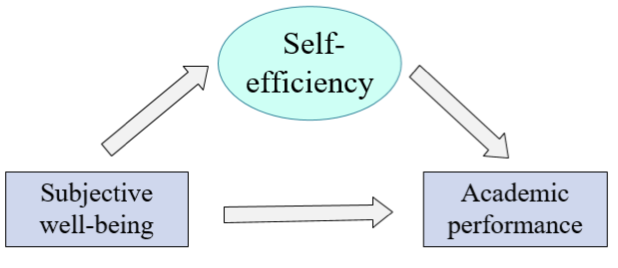
Relationship between learning self-efficacy, subjective well-being, and academic performance.

Building on Bandura’s social cognitive theory, which posits that behavior arises from interactions between personal factors and environmental influences. “Bandura’s social cognitive theory posits that human behavior is shaped by the triadic reciprocal determinism among personal factors (e.g., beliefs, emotions), behavioral patterns (e.g., learning strategies), and environmental influences (e.g., family, school). Grounded in this framework, our study conceptualizes subjective well-being (personal factor), learning self-efficacy (behavioral mediator), and academic performance (environmentally evaluated outcome) as dynamically interacting components. This study focuses on learning self-efficacy as a potential mediator. Prior research indicates that children with elevated subjective well-being exhibit stronger learning self-efficacy, which in turn enhances academic persistence and achievement. However, the specific pathways through which subjective well-being affects performance—directly or via learning self-efficacy—remain underexplored. Consequently, this study endeavors to explore the interrelations between subjective well-being and academic performance in children and to assess the mediating role of learning self-efficacy in this dynamic, thereby contributing insights to the enhancement of educational approaches and the promotion of mental health development among children. In this study, subjective well-being was measured using a multi-dimensional scale assessing life satisfaction and emotional experiences, while learning self-efficacy was evaluated through a scale focusing on children’s beliefs in their capabilities to perform academic tasks. Academic performance was gaged by both ordinary performance and test performance indicators. The relationship between the three variables is shown in Figure 1.

## 2 Literature review

### 2.1 Hypothesis of the relationship between children’s subjective well-being and learning self-efficacy

Individuals aged between 13 and 18 years are generally classified as teenagers. This phase signifies a transitional period characterized by evolving personality, interests, and learning capabilities, marking the progression from a sheltered childhood to autonomous adulthood. Academically, teenagers primarily encompass middle and high school students, with middle school acknowledged as a particularly pressurized phase, wherein familial and educational institutions wield significant influence. An imbalanced focus on academic achievements, neglecting the holistic development, can potentially precipitate mental health issues in teenagers. Excessive restrictions can induce heightened stress levels, thereby influencing academic performance. Thus, there exists an intrinsic link between children’s academic performance and their mental state, which can be gaged through subjective well-being. Subjective well-being denotes individuals’ comprehensive evaluation of their life quality based on self-determined standards.

Subjective well-being in children can be assessed from multiple perspectives, thereby yielding diverse measurement methods (Xing et al., 2022; Lei et al., 2020). Firstly, life satisfaction denotes children’s appraisal of their life quality and contentment, measured by Neugarten’s et al. (1961) Life Satisfaction Index Scale (LSI) (Nabunya et al., 2022). Secondly, the perspective of mental health posits that happiness in teenagers is manifested in mood and emotion, with corresponding measurement methods such as the Bradburn emotional scale (Zyberaj, 2022). Thirdly, a robust mental health foundation is essential for perceived happiness. Fourthly, the psychological development perspective suggests that content teenagers are more likely to realize their potential and value. Fifthly, from a self-assessment standpoint, subjective happiness perception is children’s individual appraisal of life quality and emotional response.

These diverse perspectives underscore that the subjective well-being of children is predominantly influenced by the discrepancy between their behavioral outcomes and objectives. A diminished gap correlates with elevated subjective well-being. Consequently, a correlation is postulated between children’s subjective well-being and learning self-efficacy. Learning self-efficacy refers to individuals’ subjective assessment of their capacity to accomplish specific tasks, reflective of their self-confidence. Four primary factors influence adolescent learning self-efficacy. The first is past experiences, wherein prior successes or failures shape self-evaluation and subsequent learning self-efficacy. Secondly, external evaluations and encouragements, particularly from educators, bolster self-confidence, provided they are realistic. Thirdly, physiological emotional arousal impacts task engagement and learning self-efficacy, with moderate emotions being beneficial and excessive emotions detrimental. Fourthly, vicarious experiences, gleaned from observing others, form efficacy expectations. In essence, learning self-efficacy levels, influenced by task difficulty, individual effort, external assistance, and situational context, determine task persistence and goal attainment. Elevated learning self-efficacy corresponds with higher achievement and subjective well-being, while lower levels indicate potential difficulties in task completion and diminished well-being. Consequently, subjective well-being is posited to exert a decisive influence on children’s learning self-efficacy.

In light of the aforementioned theoretical examination and extant empirical research, the ensuing research hypotheses are proposed:

*H1:* Children’s subjective well-being has a positive effect on learning self-efficacy.

### 2.2 Hypothesis of the relationship between children’s learning self-efficacy and academic performance

Adolescent academic performance serves as a significant indicator reflecting their learning attitudes, abilities, and acquisitions over a designated timeframe, constituting a pivotal basis for parental and educator involvement in adolescent learning and life experiences (Kim and Park, 2020; Wang et al., 2021). Academic performance in teenagers is bifurcated into immediate academic indicators, such as classroom performance, and prolonged learning outcomes, termed as academic achievement, exemplified by examination results. It is imperative for parents and educators to facilitate optimal academic achievement in children by nurturing and augmenting their immediate academic performance. Academic performance represents a noteworthy behavioral outcome for children, with attaining commendable academic achievement being a paramount behavioral objective. Consequently, a correlation between academic performance and children’s subjective well-being is hypothesized.

Simultaneously, children’s academic outcomes are derived from their objective endeavors, aligned with specific objectives. For instance, final examinations evaluate the accumulated knowledge over a semester, prompting teenagers to appraise their capabilities in objective practices, thus determining their potential achievements. Conversely, a deficiency in self-confidence implies a diminished ability to actualize their potential, suggesting a probable correlation between children’s academic outcomes and their level of learning self-efficacy.

In an expansive context, children’s academic performance delineates their learning proficiency, explicitly exhibited through language articulation, listening comprehension, written expression, computational abilities, logical reasoning, and classroom participation, among others (Mishra, 2022). It embodies the essence of their comprehensive learning experiences. Children’s learning proficiency is influenced by both their physiological and psychological well-being. Physiologically, the level of physical health and intellectual development significantly impacts their academic outcomes. Psychologically, aspects such as self-confidence, self-esteem, interest, and ambition play a pivotal role in modulating academic performance, with learning self-efficacy, represented by self-confidence, exerting a particularly noteworthy impact.

The level of learning self-efficacy is contingent upon task difficulty, individual effort, external assistance received, and the context of achievement realization. Correspondingly, adolescent academic performance is intimately connected to academic challenge, individual endeavor, educational support received, and the aptness of evaluative methods. Generally, increased study difficulty, augmented individual effort, enhanced educational support, and suitable evaluative methods tend to elevate children’s level of learning self-efficacy, thereby improving their academic performance. Consequently, a positive correlation between the level of learning self-efficacy and academic performance in children is plausible.

Furthermore, the level of learning self-efficacy also dictates children’s perseverance in task completion. For children’s demonstrating commendable academic performance, the learning trajectory is consistent and sustained, necessitating the maintenance of learning self-efficacy above a certain threshold. Conversely, for those exhibiting suboptimal academic performance, the learning trajectory is sporadic, and achieving elevated levels of learning self-efficacy becomes challenging. Hence, the level of learning self-efficacy emerges as a crucial determinant influencing adolescent academic performance.

Building upon the preceding theoretical elucidation and extant empirical research findings, subsequent research hypotheses are hereby proposed:

*H2:* Adolescent learning self-efficacy has a positive effect on academic performance.

### 2.3 Influence of subjective well-being on academic performance of children

From the above analysis, it can be seen that there is a certain relationship between children’s subjective well-being and learning self-efficacy, and the hypothesis “*H1*: Children’s subjective well-being has a positive effect on learning self-efficacy” is proposed, and there is also a certain relationship between children’s learning self-efficacy and academic performance,” and “*H2*: Children’s learning self-efficacy has a positive effect on academic performance” is proposed. Therefore, it can be found that the correlation between children’s subjective well-being and academic performance can be established through learning self-efficacy. Therefore, the following research hypothesis is proposed:

*H3:* Children’s subjective well-being has a positive effect on their learning performance.

*H4:* Adolescent learning self-efficacy plays a mediating role between subjective well-being and learning performance.

Based on the above analysis, the following specific research objectives are proposed:

1. To explore the differences in subjective well-being, learning self-efficacy, and academic performance between different demographic groups (e.g., gender, grade level).
2. To investigate the impact of family structure and upbringing on subjective well-being and learning self-efficacy.
3. To examine the influence of school resources and environment on academic performance.

These objectives aim to provide a comprehensive understanding of the factors affecting children’s academic performance and their interrelationships.

## 3 Methodology

### 3.1 Research objects

To ensure the diversity and representativeness of the study participants, we conducted a questionnaire survey across seven schools located in seven relatively independent areas of City A. Prior to data collection, a sample size estimation was performed using G*Power 3.1, based on an expected medium effect size (Cohen’s *f*^2^ = 0.15), *α* = 0.05, and power = 0.80, which yielded a minimum required sample size of 850 participants. Our final sample (1,022 valid responses) exceeded this threshold. The study protocol was reviewed and approved by the Ethics Committee of Jiangnan University (Approval No. JNU-2023-EC-045). Informed consent was obtained from all participants, and for minors, parental consent was also secured. Participants were informed about the purpose of the study, their right to withdraw at any time, and the confidentiality of their responses. All data were anonymized and stored securely to protect participant privacy.

Data collection procedures: The questionnaires were administered in a classroom setting under the supervision of trained research assistants. Participants were given clear instructions on how to complete the questionnaire, and they were assured that their responses would remain confidential. To ensure data quality, research assistants were present to answer any questions and to ensure that participants completed the questionnaires independently. The data collection process took approximately 30–40 min per session. After data collection, the questionnaires were checked for completeness, and any incomplete or duplicate responses were excluded from the analysis.

Inclusion criteria: Participants were (1) middle or high school students aged 13–18 years (mean age = 15.2 years, SD = 1.5); (2) voluntarily agreed to participate with informed consent (parental consent for minors); (3) able to complete the questionnaire independently. Exclusion criteria: (1) Incomplete or duplicate responses; (2) students with diagnosed severe mental health disorders (e.g., clinical depression) as reported by school counselors; (3) non-native language speakers unable to comprehend the survey items.

The selected schools spanned urban (*n* = 4) and rural (*n* = 3) districts, covering varying socioeconomic statuses (low-income: 2 schools; middle-income: 3 schools; high-income: 2 schools). This stratification ensured that the sample broadly represented adolescents in City A, though generalizability to other regions requires caution due to localized socioeconomic and cultural contexts.

Recruitment process: Participants were recruited through collaboration with school administrators and teachers. Information about the study was distributed to students and their parents, and consent forms were provided. Students who met the inclusion criteria and provided informed consent (and parental consent for minors) were included in the study. Special arrangements were made to ensure that students with disabilities or language barriers could participate, including providing additional time and assistance to complete the questionnaire.

Regarding the specific characteristics of the participants, our sample included 654 male students (64%) and 368 female students. In terms of grade level distribution, there were 136 junior high school freshmen (13.3%), 223 junior high school students in middle grades (21.8%), 121 junior high school seniors (11.8%), 286 senior high school freshmen (28.0%), 197 senior high school students in middle grades (19.3%), and 59 senior high school seniors (5.8%). Regarding family background, 913 individuals (89.3%) were only children, while 109 (10.7%) were not. In terms of upbringing, 762 (74.6%) were raised by both parents, and 260 (25.4%) were not. Geographically, 694 participants (67.9%) were from urban areas, and 328 (32.1%) were from rural regions. These sample characteristics facilitated a deeper understanding of the relationship between subjective well-being and academic performance among children from diverse social and educational backgrounds.

Ultimately, we collected 1,247 questionnaires, and after careful screening to exclude duplicates and invalid responses, we obtained 1,022 valid questionnaires. The sample included 654 male students (64%) and 368 female students. In terms of family background, 913 individuals (89.3%) were only children, while 109 (10.7%) were not. In terms of upbringing, 762 (74.6%) were raised by both parents, and 260 (25.4%) were not. Geographically, 694 participants (67.9%) were from urban areas, and 328 (32.1%) were from rural regions. These sample characteristics facilitated a deeper understanding of the relationship between subjective well-being and academic performance among children from diverse social and educational backgrounds.

### 3.2 Research tools

The questionnaire used in this article consists of two parts. The first part is the basic information of the students, involving the gender, grade, family background and so on. The second part is the scale used for measurement.

#### 3.2.1 Adolescent subjective well-being scale

The subjective well-being scale used in this study has been validated in previous research involving adolescent populations. For instance, Huebener’s Multi-dimensional Student Life Satisfaction Scale (MSLSS) and Diener’s Subjective Well-being Scale for International College students (D’Souza et al., 2020; Liu et al., 2018) which served as the basis for our scale, have been widely used and validated in various cultural and educational contexts. On this basis, this study develops a scale of adolescent subjective well-being applicable to adolescent students, which is divided into two parts: life satisfaction scale and emotional experience scale. Among them, life satisfaction includes six dimensions, namely friendship, family, school, study, entertainment and environment. A total of 36 questions are designed. The scale adopts a seven-point scale. The Cronbanch’s *α* coefficient of the scale was 0.952, the KMO coefficient of appropriateness was 0.966, and the Bartlett test *p* value was 0.000, indicating that the scale had high reliability and validity and could better measure adolescent subjective well-being. The sum of the scores of each dimension is the total score of the life satisfaction scale. The higher the score of the scale, the higher the life satisfaction of the individual. The scale of emotional experience includes two dimensions of positive emotion and negative emotion. A total of 10 questions are designed, with 5 questions for each dimension. The sum of scores of each dimension is the scale of emotional experience.

Sample items for each subscale are as follows:

Life satisfaction (alpha = 0.945): “I am satisfied with my life,” “I enjoy spending time with my friends,” “My family supports me in my endeavors,” “I feel safe and comfortable at school,” “I find my studies engaging and fulfilling,” “I have access to entertainment activities that I enjoy,” “I feel comfortable and secure in my environment.”

Positive emotion (alpha = 0.892): “I feel happy,” “I experience joy in my daily activities,” “I feel optimistic about the future.”

Negative emotion (alpha = 0.876): “I feel sad,” “I experience anxiety in stressful situations,” “I feel discouraged when facing difficulties.”

#### 3.2.2 Learning the learning self-efficacy scale

The learning self-efficacy scale employed in this study draws on established frameworks that have been utilized in previous research. Specifically, the dimensions of learning capacity, learning willingness, and learning behavior have been adapted from scales used in educational psychology studies to measure academic learning self-efficacy. This learning self-efficacy scale is divided into three dimensions: learning capacity, learning willingness and learning behavior (Quan et al., 2016; Tu and Zhang, 2015). Learning self-efficacy of learning ability refers to the judgment and evaluation of children’s belief in their ability to complete academic tasks, while learning self-efficacy of will to learn refers to the judgment and evaluation of children’s belief in their ability to persist in completing learning tasks. Learning self-efficacy of learning behavior refers to teenagers’ judgment and evaluation of how much they believe in learning methods and measures to complete tasks and achieve their learning goals. A total of 24 questions were designed, 8 questions for each aspect, using a five-point scoring method, ranging from completely agree to completely disagree. The Cronbanch’s *α* coefficient of the scale was 0.946, the KMO sampling appropriateness coefficient was 0.968, and the Bartlett test *p* value was 0.000, indicating that the scale had high reliability and validity and could better measure the academic learning self-efficacy of children. The sum of the scores of the three dimensions of the scale is the total score of academic learning self-efficacy. The higher the scale score, the higher the individual learning self-efficacy.

Sample items for each subscale are as follows:

Learning capacity (alpha = 0.934): “I believe I can understand complex academic concepts” and “I am confident in my ability to complete assignments accurately.”

Learning willingness (alpha = 0.912): “I am motivated to continue working on difficult tasks” and “I am determined to achieve my academic goals.”

Learning behavior (alpha = 0.908): “I can effectively organize my study time” and “I know how to use different learning strategies to improve my performance.”

#### 3.2.3 Academic performance scale

The academic performance scale in this study has been expanded to encompass a broader range of indicators that reflect the multifaceted nature of students’ educational experiences. This comprehensive evaluation method was chosen as it provides a multi-dimensional assessment of students’ academic capabilities, capturing both their day-to-day performance and achievements across various aspects of learning. By incorporating ordinary performance, test performance, and comprehensive competence, the scale offers a holistic view of academic performance that aligns with contemporary educational goals and standards. The scale now includes three main levels: ordinary performance, test performance, and comprehensive competence. Each level is further divided into specific dimensions that provide a more detailed assessment of students’ academic performance.

1. Ordinary performance

Classroom Interaction: Assesses the quality and frequency of student participation in classroom activities. The scale ranges from 1 (“no participation at all”) to 5 (“active participation”).

Homework: Evaluates the consistency and quality of completed assignments, with ratings from 1 (“bad”) to 5 (“very good”).

1. Test performance

The assessment of academic performance in core subjects such as Chinese, mathematics, and English, using a five-point scale ranging from 1 (“bad”) to 5 (“very good”), to reflect a comprehensive evaluation of academic proficiency.

1. Comprehensive competence

Learning skills: Assesses the student’s ability to employ various learning strategies such as summarizing information, using mnemonic devices, and self-explaining concepts, which are critical for enhancing comprehension and retention of knowledge.

Motivation: Evaluates the student’s internal drive to learn and the external factors that stimulate their academic pursuits. This includes the assessment of goal orientation, the pursuit of mastery, and the response to rewards and recognition.

Student engagement: Reflects the student’s active involvement in classroom activities, including attendance, participation in discussions, and adherence to classroom routines. This dimension also considers the student’s overall presence and alertness during instructional time.

While school grades are a common metric for academic performance, they may not fully capture the complexity of a student’s learning experience and capabilities. The chosen evaluation method complements school grades by incorporating additional dimensions such as learning skills, motivation, and student engagement, which are critical for understanding the factors that influence academic success. This approach provides a more nuanced assessment that can better inform educational interventions and support strategies.

These additional aspects of Comprehensive Competence provide a more nuanced understanding of a student’s academic profile, highlighting the importance of not just cognitive abilities but also the motivational and behavioral factors that contribute to overall academic success. The respondents complete the questionnaire based on their self-perception of these various aspects of academic performance. The total score for each dimension represents the academic performance score, with higher scores indicating better self-evaluation of academic competence. The scale’s reliability is supported by a Cronbach’s alpha of 0.85, indicating good internal consistency. The KMO sampling appropriateness coefficient is 0.90, and the Bartlett’s test of sphericity yielded a *p* value of 0.000, confirming the suitability of the scale for the analysis and its ability to comprehensively measure the different dimensions of academic performance.

Sample items for each subscale are as follows:

Classroom Interaction (alpha = 0.895): “I actively participate in class discussions,” “I ask questions when I do not understand a concept.”

Homework (alpha = 0.887): “I complete my homework on time and to a high standard,” “I review my homework to ensure accuracy.”

Test Performance (alpha = 0.879): “I perform well on exams in core subjects,” “I feel prepared for tests and assessments.”

Learning Skills (alpha = 0.864): “I can summarize information effectively,” “I use mnemonic devices to remember important details.”

Motivation (alpha = 0.853): “I am intrinsically motivated to learn new things,” “I set academic goals for myself and work towards them.”

Student Engagement (alpha = 0.847): “I attend classes regularly,” “I pay attention during instructional time.”

#### 3.2.4 Assessment of school resources

School resources were assessed using a composite measure based on data provided by school administrators. The measure included the following indicators:

1. Number of teachers per student: This indicator reflects the teacher-to-student ratio, which is a key factor in the quality of education.
2. Availability of educational materials: This includes the availability of textbooks, laboratory equipment, and other learning resources.
3. Infrastructure quality: This includes the condition of school buildings, classrooms, and recreational facilities.
4. Extracurricular activities: The availability and variety of extracurricular programs offered by the school.

Data on these indicators were collected through a survey administered to school administrators. Each indicator was rated on a 5-point scale, with higher scores indicating better resources. The overall school resources score was calculated as the average of the scores for all indicators. This measure was used to assess the relationship between school resources and students’ academic performance.

#### 3.2.5 Statistical analysis methods and control of confounding variables

All statistical analyses were conducted using SPSS 26.0 (IBM Corp., Armonk, NY), including descriptive statistics, multivariate regression, and mediation analysis via Hayes’ PROCESS macro (Version 3.5). For mediation analysis, we used the PROCESS macro (Version 3.5) developed by Hayes, which is a widely used tool for testing mediation and moderation effects in SPSS. To control for potential confounding variables, we utilized multivariate regression models during the data analysis phase. Specifically, we included demographic variables such as age, gender, family structure (e.g., only-child status), and parental education level as control variables in our models. Additionally, we considered school-level factors such as resource availability and teacher quality through multilevel models (hierarchical linear modeling) to account for the nested structure of students within schools. The models included variables such as students’ age, gender, and family background (e.g., whether they were only children, the level of their parents’ education). Additionally, we considered the effects of students’ grade levels to reflect the pressures and challenges that different academic stages might bring.

Given that students’ grades and behaviors may be influenced by the school environment, we employed multilevel models to analyze the data, addressing the nested hierarchical structure of students within schools. This type of model allowed us to differentiate factors at the individual level (such as students’ personal characteristics) from those at the school level (such as school resources and teacher quality). Through this analysis, we were able to assess the potential impact of the school environment on academic performance. For instance, we found a significant positive correlation between the richness of school resources and students’ academic performance (*r* = 0.35, *p* < 0.01), a finding that underscores the importance of school-level factors in academic achievement.

## 4 Research results and discussions

### 4.1 Differences in children’s subjective well-being, learning self-efficacy and academic performance

The descriptive statistics and independent sample *T*-test of the subjective well-being of teenagers in middle and high schools and its dimensions are presented. The statistical and calculation results are shown in Table 1. The statistical tests used for comparisons in Table 1 were independent samples *T*-tests and ANOVA. ANOVA is a versatile statistical method that can handle both two-group and multi-group comparisons, and it provides a consistent framework for analyzing differences across various variables. Additionally, ANOVA allows us to conduct post-hoc tests if needed, which can be useful for exploring complex patterns in the data. While a *t*-test would have been appropriate for comparing two groups, ANOVA offered a more flexible and unified approach to our data analysis, particularly when dealing with multiple dependent variables and dimensions. The *T*-tests were utilized to compare the mean scores of subjective well-being, learning self-efficacy, and academic performance between male and female students, as well as between junior and senior high school students. ANOVA was employed to detect any significant differences between junior high school and senior high school students. Regarding clinical significance, the differences found in subjective well-being scores between various groups (e.g., junior vs. senior high school students, different family structures) were statistically significant but may not necessarily translate to clinically significant differences. The effect sizes and practical implications of these differences should be considered in the context of educational interventions and support strategies.

**Table 1:** Descriptive statistics results and *T*-test results.

Items	Dimensions	Total points	Junior		Senior		t	p
			M	SD	M	SD		
Subjective well-being	Positive	35 points	26.12	7.82	24.36	7.31	3.42	0.001
	Passive	35 points	27.2	7.54	26.34	7.12	2.01	0.205
	Friendship	42 points	31.06	6.65	30.86	6.13	1.12	0.921
	Family	42 points	32.16	7.46	33.02	7.13	1.01	0.469
	School	42 points	30.15	8.13	28.97	8.46	2.87	0.000
	Academy	42 points	23.68	7.19	22.16	6.85	1.630	0.099
	Entertainment	42 points	22.76	6.17	22.10	6.03	1.09	0.287
	Environment	42 points	26.54	4.36	25.47	4.83	2.43	0.010
	Total	322 points	219.67	36.86	213.28	35.42	3.12	0.003
Learning self-efficacy	Learning capacity	40 points	23.26	8.03	22.95	9.35	2.04	0.004
	Learning willingness	40 points	21.37	7.65	20.67	6.84	2.16	0.001
	Learning behavior	40 points	20.69	7.34	20.13	7.13	4.36	0.000
	Total	120 points	65.32	13.68	62.75	11.36	4.10	0.000
Academic performance	Interaction in classroom	5 points	3.62	4.69	3.83	4.01	1.36	0.003
	Homework	5 points	3.84	2.89	4.15	3.25	1.78	0.000
	Test performance	5 points	3.16	3.67	3.27	3.64	1.72	0.001
	Total	15 points	10.62	3.23	11.25	3.14	1.83	0.000
Comprehensive competence	Learning skills	10 points	7.58	2.45	7.12	2.21	2.47	0.014
	Motivation	10 points	8.45	2.58	7.98	2.46	2.56	0.011
	Student engagement	10 points	7.61	2.19	7.29	2.03	2.17	0.03
	Total	30 points	23.64	7.22	22.39	6.7	7.2	0.055

It can be seen from the data in Table 1 that there is no significant difference between teenagers in middle school and high school in the five dimensions of negative emotion, friendship, family, study and entertainment (*p* > 0.05), but there is a significant difference in the environmental dimension of scores (*p* < 0.05), and the total score of subjective well-being has a very significant difference (*p* < 0.01). There was a significant difference in the score of positive emotion and school dimension. It can be seen that the overall level of subjective well-being of teenagers in middle school and high school is in the upper class. There is no significant difference between junior high school students and senior high school students in the dimensions of negative emotion, friendship, family, study and entertainment, but there are great differences in the dimensions of positive emotion, environment and school, that is to say, positive emotion, school and environment and other factors are the main reasons for the lower subjective well-being of senior high school students than junior high school students.

Descriptive statistics and independent sample *T*-test were conducted on the learning self-efficacy and its dimensions of teenagers in middle and high schools. The statistical and calculation results were shown in Table 1. As can be seen from Table 1, there are significant differences (*p* < 0.05) between junior high school and senior high school children in the scores of learning capacity learning self-efficacy, learning willingness learning self-efficacy and learning behavior learning self-efficacy, and the scores of junior high school students are higher than those of senior high school students. At the same time, the difference of learning self-efficacy between junior high school students and senior high school students mainly comes from learning will and learning behavior. For instance, the Cohen’s d for learning self-efficacy between junior and senior high school students was 0.26, indicating a medium effect size.

Descriptive statistics and independent sample *T*-test were conducted on the academic performance and its dimensions of teenagers in middle and high schools. The statistical and calculation results were shown in Table 1. As can be seen from Table 1, there are extremely significant differences between junior high school and senior high school teenagers in their usual performance and exam performance (*p* < 0.01), and junior high school students score lower than senior high school students. At the same time, the difference in academic performance between middle and high school students is mainly due to homework. The Eta square for academic performance differences between junior and senior high school students was 0.032, suggesting a small to medium effect size.

### 4.2 Teenagers’ subjective well-being, learning self-efficacy and academic performance are affected by other factors

#### 4.2.1 Preliminary analysis of variables by gender and level of study

Before testing the hypotheses, we conducted a preliminary analysis to explore the differences in subjective well-being, learning self-efficacy, and academic performance according to gender and level of study (junior vs. senior). This analysis provides a foundation for understanding the underlying patterns that may influence the relationships proposed in our hypotheses.

We utilized independent samples *t*-tests to compare the mean scores of subjective well-being, learning self-efficacy, and academic performance between male and female students. Additionally, we employed ANOVA to detect any significant differences between junior high school and senior high school students.

The *t*-tests indicated that female students reported higher levels of subjective well-being (*M* = 4.5, SD = 0.8) compared to male students (*M* = 4.2, SD = 0.9), with a significant difference observed (*t*(1020) = 3.45, *p* < 0.01). Male students demonstrated higher learning self-efficacy scores (*M* = 3.8, SD = 0.7) than female students (*M* = 3.5, SD = 0.8), which also reached significance (*t*(1020) = 4.75, *p* < 0.001).

Regarding the level of study, senior high school students showed a higher sense of learning self-efficacy (*M* = 4.0, SD = 0.6) compared to junior high school students (*M* = 3.7, SD = 0.5), as indicated by a significant *F*-value (*F*(1, 1,020) = 15.83, *p* < 0.001). Conversely, junior high school students reported greater satisfaction in the friendship domain (*M* = 4.8, SD = 0.4) than senior high school students (*M* = 4.5, SD = 0.5), with a significant difference (*F*(1, 1,020) = 9.42, *p* < 0.01).

These preliminary findings suggest that gender and educational level are factors that may influence children’s subjective well-being and learning self-efficacy. The observed differences provide a basis for further exploration and understanding of the underlying mechanisms proposed in the hypotheses.

#### 4.2.2 Influence of demographic and family factors on Children’s well-being and academic outcomes

Descriptive statistics and independent sample *T*-test were conducted on the subjective well-being, learning self-efficacy and academic performance of children in junior and senior middle schools of different genders. The statistical and calculation results are shown in Table 2. As can be seen from Table 2, the learning self-efficacy dimension of the junior middle school group is greatly affected by gender, while the subjective well-being dimension of the senior middle school group is greatly affected by gender.

**Table 2:** Descriptive statistics results under the influence of other factors.

Dimensions		Subjective well-being		Learning self-efficacy		Academic performance	
		Junior	Senior	Junior	Senior	Junior	Senior
Male	M	224.83	225.12	65.42	63.15	10.44	11.21
	SD	33.16	30.45	14.11	11.25	3.22	2.83
Female	M	223.67	226.47	64.87	62.98	10.12	10.58
	SD	30.25	32.13	13.24	12.09	2.87	3.86
*t*		2.58	1.98	1.68	1.73	2.14	2.67
*p*		0.122	0.045	0.039	0.101	0.133	0.072
Only child	M	225.96	223.47	66.81	61.53	10.98	11.65
	SD	35.26	31.32	15.35	12.49	3.78	3.12
Not only child	M	217.87	211.63	65.38	61.03	10.53	10.69
	SD	30.49	34.85	14.22	11.97	3.13	3.24
*t*		2.69	2.46	1.95	1.87	2.01	1.89
*p*		0.008	0.020	0.057	0.113	0.000	0.005
Raised by both parents	M	224.48	221.63	69.84	65.37	11.46	12.03
	SD	30.24	28.35	13.45	11.02	3.21	2.84
Raised not by both parents	M	212.38	203.54	58.21	54.32	9.86	10.32
	SD	30.24	28.35	16.34	17.24	4.51	5.06
*t*		2.79	2.14	3.56	3.24	1.32	−3.21
*p*		0.000	0.000	0.000	0.000	0.164	0.000
From city	M	223.89	220.77	68.21	62.11	11.32	11.87
	SD	28.24	27.64	13.21	11.47	2.25	1.36
From county	M	214.03	207.14	57.02	55.14	9.65	10.03
	SD	27.63	28.87	14.36	13.79	2.67	2.35
*t*		1.58	−0.46	1.22	−0.98	1.22	1.03
*p*		0.087	0.825	0.608	0.883	0.143	0.387

Descriptive statistics and independent sample *T*-test were conducted on the subjective well-being, learning self-efficacy and academic performance of the teenagers in the junior and senior middle school groups of only children and non-only children. The statistical and calculation results are shown in Table 2. As can be seen from Table 2, the subjective well-being and learning self-efficacy of teenagers in the middle school group and the high school group are significantly affected by whether they are the only child or not, and the academic performance is extremely significantly affected. The Cohen’s d for subjective well-being between only children and non-only children was 0.32, indicating a medium effect size. The Eta square for academic performance differences was 0.045, suggesting a medium effect size.

We found a significant difference between only children and non-only children in terms of subjective well-being after controlling for other variables (*t*(1020) = 2.69, *p* = 0.008). The mean subjective well-being score for only children was 225.96 (SD = 35.26), while the mean score for non-only children was 217.87 (SD = 30.49). This indicates that only children reported higher levels of subjective well-being compared to non-only children.

Descriptive statistics and independent sample *T*-test were conducted on the subjective well-being, learning self-efficacy and academic performance of children in the junior and senior middle school groups who are jointly raised by their parents. The statistical and calculation results are shown in Table 2. As can be seen from Table 2, the subjective well-being and learning self-efficacy of children in the middle school and high school groups are significantly affected by family integrity, and the scores of children from non-parental families are lower.

Descriptive statistics and independent sample *T*-test were conducted on the subjective well-being, learning self-efficacy and academic performance of the teenagers from the middle school group and the high school group whose family backgrounds were urban and rural, respectively. The statistical and calculation results were shown in Table 2. As can be seen from Table 2, family background has a significant impact on the subjective happiness of teenagers in the junior middle school group, while other variables are not significantly affected by family background.

### 4.3 Analysis of the correlation between children’s subjective well-being, learning self-efficacy and academic performance

Pearson product correlation analysis was conducted on the subjective well-being, learning self-efficacy and academic performance of children in the junior middle school group, and the results were shown in Table 3. As can be seen from Table 3, junior high school students’ subjective well-being and its various dimensions have significant correlation with academic performance. The separate analysis for junior and senior high school students revealed nuanced differences in the relationships between subjective well-being, learning self-efficacy, and academic performance at different educational stages. For junior high school students, the correlation coefficient between subjective well-being and academic performance was 0.343 (*p* < 0.01), indicating a moderate positive relationship. For senior high school students, this correlation was slightly higher at 0.351 (*p* < 0.01). These findings suggest that while the overall patterns are consistent, the strength of these relationships may vary with developmental stage and academic context. Additionally, the correlation between subjective well-being and learning self-efficacy was 0.489 for junior high school students and 0.477 for senior high school students, both of which are statistically significant and indicate strong positive relationships. This further underscores the importance of considering educational stage when designing interventions aimed at enhancing academic outcomes through improvements in subjective well-being and learning self-efficacy.

**Table 3:** Descriptive statistics of children in junior based on Pearson product difference correlation analysis.

	1	2	3	4	5	6	7	8	9	10	11	12	13	14
1 Positive	1													
2 Passive	0.126*	1												
3 Friendship	0.382**	0.328**	1											
4 Family	0.362**	0.297**	0.401**	1										
5 School	0.457**	0.355**	0.432**	0.382**	1									
6 Academy	0.346**	0.346**	0.446**	0.376**	0.359**	1								
7 Entertainment	0.378**	0.413**	0.501**	0.342**	0.368**	0.322**	1							
8 Environment	0.400**	0.384**	0.531**	0.359**	0.328**	0.421**	0.413**	1						
9 Subjective well-being	0.592**	0.588**	0.623**	0.551**	0.501**	0.509**	0.566**	0.551**	1					
10 Learning capacity	0.371**	0.311**	0.423**	0.374**	0.402**	0.411**	0.397**	0.416**	0.456**	1				
11 Learning willingness	0.365**	0.242**	0.388**	0.392**	0.364**	0.376**	0.387**	0.399**	0.403**	0.377**	1			
12 Learning behavior	0.369**	0.302**	0.394**	0.403**	0.383**	0.403**	0.416**	0.402**	0.422**	0.368**	0.433**	1		
13 Learning self-efficacy	0.403**	0.376**	0.436**	0.476**	0.461**	0.458**	0.448**	0.439**	0.489**	0.461**	0.453**	0.496**	1	
14 Academic performance	0.246**	0.201**	0.310**	0.311**	0.298**	0.186**	0.214**	0.325**	0.343**	0.302**	0.248**	0.221**	0.188**	1
*M*	20.32	38.23	36.45	37.44	36.22	37.12	28.96	26.83	32.14	30.41	29.84	20.46	21.63	10.33
SD	7.012	7.113	6.885	7.200	7.334	7.453	8.124	5.269	6.745	7.134	6.324	5.362	5.246	2.351

**p < 0.01, *p < 0.05.

Pearson product correlation analysis was conducted on the subjective well-being, learning self-efficacy and academic performance of children in the junior middle school group, and the results were shown in Table 4. As can be seen from Table 4, high school students’ subjective well-being and its various dimensions are significantly correlated with academic performance. The correlation coefficient between the total score of subjective well-being and academic performance is 0.351, indicating that the higher the score of subjective well-being, the better the academic performance. The correlation between subjective well-being and its dimensions and learning self-efficacy and its dimensions is significant, and the correlation coefficient is 0.477, indicating that the higher the score of subjective well-being, the higher the learning self-efficacy.

**Table 4:** Descriptive statistics of children in senior based on Pearson product difference correlation analysis.

	1	2	3	4	5	6	7	8	9	10	11	12	13	14
1 Positive	1													
2 Passive	0.146*	1												
3 Friendship	0.391**	0.318**	1											
4 Family	0.354**	0.303**	0.389**	1										
5 School	0.425**	0.324**	0.412**	0.376**	1									
6 Academy	0.418**	0.316**	0.425**	0.344**	0.333**	1								
7 Entertainment	0.369**	0.395**	0.465**	0.371**	0.347**	0.342**	1							
8 Environment	0.424**	0.367**	0.481**	0.347**	0.316**	0.412**	0.393**	1						
9 Subjective well-being	0.627**	0.602**	0.635**	0.545**	0.538**	0.523**	0.536**	0.541**	1					
10 Learning capacity	0.325**	0.325**	0.412**	0.388**	0.409**	0.401**	0.383**	0.420**	0.446**	1				
11 Learning willingness	0.357**	0.248**	0.378**	0.364**	0.353**	0.387**	0.403**	0.389**	0.416**	0.365**	1			
12 Learning behavior	0.387**	0.313**	0.387**	0.409**	0.387**	0.388**	0.421**	0.412**	0.432**	0.372**	0.418**	1		
13 Learning self-efficacy	0.421**	0.384**	0.411**	0.482**	0.454**	0.428**	0.438**	0.434**	0.477**	0.458**	0.444**	0.487**	1	
14 Academic performance	0.256**	0.212**	0.302**	0.296**	0.308**	0.169**	0.202**	0.303**	0.351**	0.316**	0.263**	0.212**	0.193**	1
*M*	22.18	36.24	34.42	36.55	35.12	36.62	27.68	25.96	30.11	28.35	27.57	22.16	23.24	10.87
SD	6.882	7.225	6.465	7.013	6.746	7.343	7.698	4.285	6.883	6.331	5.314	5.035	5.642	2.125

**p < 0.01, *p < 0.05.

### 4.4 Test of the mediating role of learning self-efficacy

To examine the direct and indirect effects of subjective well-being on academic performance through learning self-efficacy, we conducted a mediation analysis using the PROCESS macro (Model 4) in SPSS, developed by Hayes. This analysis involved several key assumptions:

Normality: The distribution of residuals for the dependent variable (academic performance) was approximately normal, as assessed by visual inspection of histograms and the Shapiro–Wilk test (*p* > 0.05).

Independence: The observations were independent, given the random sampling method and the absence of repeated measures or hierarchical clustering effects beyond those accounted for in the multilevel models.

Linearity: The relationships between subjective well-being, learning self-efficacy, and academic performance were assumed to be linear, which was supported by the Pearson correlation coefficients.

For the bootstrapping procedure, we used 1,000 bootstrap samples with a 95% confidence interval to estimate the indirect effects. This non-parametric bootstrapping method provided robust estimates of the sampling distribution of the indirect effect, allowing us to test the significance of the mediation effect without assuming a specific distribution.

The results were shown in Table 5. The results indicate that subjective well-being has a significant positive direct effect on academic performance (*B* = 0.24, *t* = 6.88, *p* < 0.001) and a significant positive indirect effect through learning self-efficacy (*B* = 0.46, *t* = 9.64, *p* < 0.001). The total effect of subjective well-being on academic performance is 0.70 (*B* = 0.24 + 0.46), indicating that the indirect effect via learning self-efficacy accounts for approximately 66% of the total effect. This indicates that subjective well-being can not only directly affect children’s academic performance, but also indirectly affect children’s academic performance through the mediating effect of learning self-efficacy. Hypothesis H4 is further supported.

**Table 5:** Mediating effect analysis of learning self-efficacy.

Path	*B*	*t*	95% CI lower	95% CI upper	Effect size
Subjective well-being → Learning self-efficacy	0.68	28.43***	0.61	0.7	97%
Subjective well-being → Academic performance (Direct)	0.24	6.88***	0.2	0.33	34%
Learning self-efficacy → Academic performance	0.46	9.64***	0.33	0.48	66%

***p < 0.001.

Additionally, the results provide strong support for the other hypotheses:

*H1:* Children's subjective well-being has a positive effect on learning self-efficacy. This hypothesis was supported by the significant positive relationship between subjective well-being and learning self-efficacy.

*H2:* Adolescent learning self-efficacy has a positive effect on academic performance. This hypothesis was supported by the significant positive relationship between learning self-efficacy and academic performance.

*H3:* Children's subjective well-being has a positive effect on their learning performance. This hypothesis was supported by the significant direct effect of subjective well-being on academic performance.

**Figure 2:**
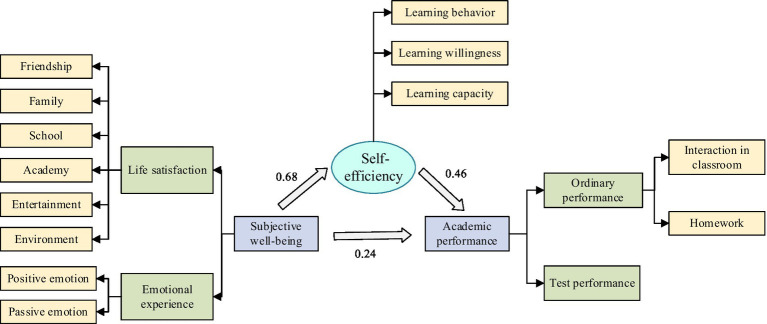
Possible relationship among subjective well-being, learning self-efficacy, and academic performance of children.

These findings collectively demonstrate that subjective well-being influences academic performance both directly and indirectly through learning self-efficacy, providing comprehensive support for all proposed hypotheses (Figure 2).

## 5 Conclusion

This study demonstrates that children’s subjective well-being positively influences academic performance through both direct and indirect pathways, with learning self-efficacy playing a pivotal mediating role. The findings reveal that subjective well-being not only directly enhances academic performance but also indirectly improves it by boosting learning self-efficacy, particularly through learning behavior and task persistence.

Consistent with Li et al. (2023), we found that life satisfaction and emotional experiences significantly predict academic performance. However, our study uniquely identifies environmental factors and family structure (e.g., only-child status) as key moderators of subjective well-being, offering new insights into the contextual influences on adolescent development. For instance, children raised in urban environments reported higher subjective well-being compared to their rural counterparts, highlighting the role of resource availability and social support.

The mediating role of learning self-efficacy aligns with Koca et al. (2024) findings but extends their work by emphasizing the behavioral dimensions of learning self-efficacy, such as learning strategies and task persistence. This suggests that interventions targeting learning self-efficacy, particularly in the context of learning behaviors, could significantly enhance academic outcomes. Additionally, our findings resonate with Diotaiuti et al. (2021), who emphasized the role of emotional balance in mitigating procrastination and improving academic performance, suggesting that emotional regulation strategies could complement learning self-efficacy interventions.

The practical implications of these findings are significant for the educational field. First, schools should prioritize mental health programs that foster subjective well-being, such as mindfulness training and emotional regulation workshops. These programs can help students manage stress and build resilience, which are critical for maintaining high levels of well-being. Second, educators should implement learning self-efficacy enhancement strategies, such as goal-setting exercises, peer mentoring programs, and scaffolded learning tasks that gradually increase in difficulty. These interventions can help students develop confidence in their academic abilities and improve their persistence in the face of challenges. Third, schools should create supportive environments that address the unique needs of students from diverse backgrounds, such as those from rural areas or non-traditional family structures. For example, providing access to counseling services and extracurricular activities can help mitigate the negative effects of environmental and familial stressors.

Despite these contributions, this study has several limitations. First, the cross-sectional design limits our ability to infer causal relationships between subjective well-being, learning self-efficacy, and academic performance. Longitudinal studies are needed to examine how these variables interact over time. Second, the reliance on self-reported measures for subjective well-being and learning self-efficacy may introduce response bias. Future research could incorporate multi-source data, such as teacher and parent evaluations, to enhance the objectivity of the findings. Finally, the sample was limited to middle and high school students from a specific region, which may affect the generalizability of the results. Replicating this study with diverse populations and longitudinal data would strengthen the validity and applicability of the findings. In future research, we will combine the evaluations of teachers and parents, adopt a multi-source information collection method to more comprehensively and objectively evaluate students’ academic performance, and deeply explore the consistency and differences between data from different sources, further improving the scientific and feasible nature of the research. In addition, we plan to use more detailed structural equation modeling in future research to explore in depth how each sub dimension uniquely affects academic performance and the differences they may have at different educational stages.

## Data Availability

The original contributions presented in the study are included in the article/supplementary material, further inquiries can be directed to the corresponding author.
